# 3,3-Bis(2-hy­droxy­eth­yl)-1-(4-methyl­benzoyl)thio­urea: crystal structure, Hirshfeld surface analysis and computational study

**DOI:** 10.1107/S2056989019012581

**Published:** 2019-09-12

**Authors:** Sang Loon Tan, Ainnul Hamidah Syahadah Azizan, Mukesh M. Jotani, Edward R. T. Tiekink

**Affiliations:** a Research Centre for Crystalline Materials, School of Science and Technology, Sunway University, 47500 Bandar Sunway, Selangor Darul Ehsan, Malaysia; b Department of Physics, Bhavan’s Sheth R. A. College of Science, Ahmedabad, Gujarat 380001, India

**Keywords:** crystal structure, thio­urea, hydrogen bonding, Hirshfeld surface analysis, computational chemistry

## Abstract

In the title tri-substituted thio­urea derivative, C_13_H_18_N_2_O_3_S, the thione-S and carbonyl-O atoms lie, to a first approximation, to the same side of the mol­ecule [the S—C—N—C torsion angle is −49.3 (2)°]. The CN_2_S plane is almost planar (r.m.s. deviation = 0.018 Å) with the hy­droxy­ethyl groups lying to either side of this plane. One hy­droxy­ethyl group is orientated towards the thio­amide functionality enabling the formation of an intra­molecular N—H⋯O hydrogen bond leading to an *S*(7) loop. The dihedral angle [72.12 (9)°] between the planes through the CN_2_S atoms and the 4-tolyl ring indicates the mol­ecule is twisted. The experimental mol­ecular structure is close to the gas-phase, geometry-optimized structure calculated by DFT methods. In the mol­ecular packing, hydroxyl-O—H⋯O(hydrox­yl) and hydroxyl-O—H⋯S(thione) hydrogen bonds lead to the formation of a supra­molecular layer in the *ab* plane; no directional inter­actions are found between layers. The influence of the specified supra­molecular inter­actions is apparent in the calculated Hirshfeld surfaces and these are shown to be attractive in non-covalent inter­action plots; the inter­action energies point to the important stabilization provided by directional O—H⋯O hydrogen bonds.

## Abstract 2

The title tri-substituted thio­urea derivative is twisted with a dihedral angle of 72.12 (9)° between the planes through the CN_2_S atoms and the 4-tolyl ring; an intra­molecular N–H⋯O hydrogen bond leads to an *S*(7) loop. In the crystal. hydroxyl-O—H⋯O(hydrox­yl) and hydroxyl-O—H⋯S(thione) hydrogen bonds give rise to a supra­molecular layer in the *ab* plane.

## Chemical context   

The amine-H atoms in thio­urea, H_2_NC(=S)NH_2_, can be systematically replaced to generate up to tetra-functionalized mol­ecules, *i.e. R* ^1^(*R* ^2^)NC(=S)N(*R* ^3^)*R* ^4^ for *R* ^1–4^ = alk­yl/aryl. The present study concerns a tri-substituted example, *i.e*. an *N*,*N*′-di(alk­yl/ar­yl)-*N*′-benzoyl­thio­urea derivative, notable for having a carbonyl group connected to the thio­urea framework. Thio­urea mol­ecules are of inter­est in themselves and as ligands for metal ions (Saeed *et al.*, 2014 ▸). The free mol­ecules, including benzoyl derivatives, are well-known to exhibit various biological properties, for example, anti-bacterial, anti-fungal and anti-viral activities as well as cytotoxicity (Hallur *et al.*, 2006 ▸; Cunha *et al.*, 2007 ▸; Saeed *et al.*, 2010 ▸; Gunasekaran *et al.*, 2017 ▸; Zhang *et al.*, 2018 ▸; Gunasekaran, Ng *et al.*, 2012 ▸). The combination of hard (oxygen) and soft (sulfur) donor atoms along with nitro­gen suggests that benzoyl­thio­ureas can function as versatile ligands to metals. Indeed, a variety of coordination modes have been observed such as monodentate-S for the neutral ligand (Saeed *et al.*, 2014 ▸; Gunasekaran, Ng *et al.*, 2012 ▸). When deprotonated, a common mode of coordination is O-,S- chelation with considerable delocalization of π-electron density over the ensuing six-membered chelate ring (Saeed *et al.*, 2014 ▸). While the motivations for preparing metal complexes of benzoyl­thio­ureas are varied, *e.g*. for anion recognition and as catalysts (Saeed *et al.*, 2014 ▸; Zhang & Schreiner, 2009 ▸; Nishikawa, 2018 ▸), there is continuing inter­est in exploring their biological potential as coordination of these ligands to metals generally enhances their biological efficacy, such as anti-cancer (Peng *et al.*, 2016 ▸; Barolli *et al.*, 2017 ▸; Jeyalakshmi *et al.*, 2019 ▸), anti-microbial (Gemili *et al.*, 2017 ▸; Binzet *et al.*, 2018 ▸; Saeed *et al.*, 2018 ▸) and anti-mycobacterium tuberculosis (Plutín *et al.*, 2016 ▸) activities. The present study was motivated by these applications and by previous structural studies (Gunasekaran *et al.*, 2017 ▸; Selvakumaran & Karvembu, *et al.*, 2011 ▸; Selvakumaran, Ng *et al.*, 2011 ▸) and the known catalytic applications of their cobalt complexes (Gunasekaran, Jerome *et al.*, 2012 ▸). Herein, the synthesis, spectroscopic characterization and X-ray crystallographic investigation of the title compound, 4-MePhC(=O)N(H)C(=S)N(CH_2_CH_2_OH)_2_, (I), are described, along with an analysis of the calculated Hirshfeld surfaces, non-covalent inter­action plots as well as a computational chemistry study.
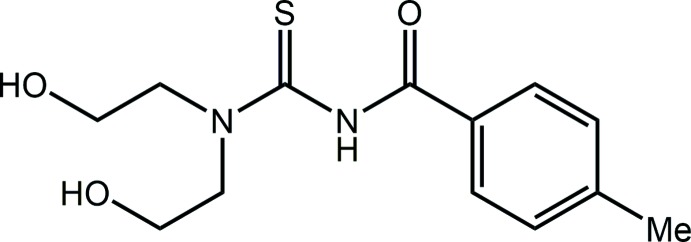


## Structural commentary   

**Figure 1:**
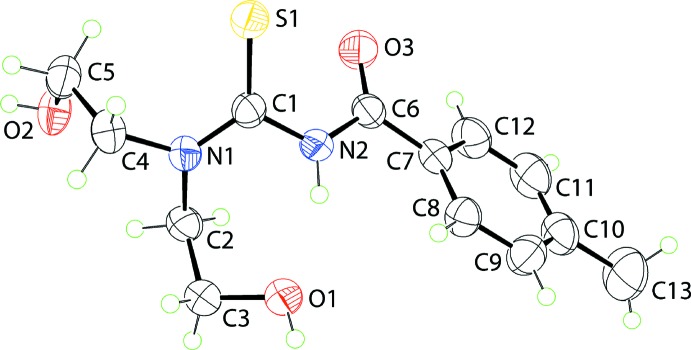
The mol­ecular structure of (I) showing the atom-labelling scheme and displacement ellipsoids at the 50% probability level.

The title compound, (I), is illustrated in Fig. 1 ▸, and selected inter­atomic parameters are given in Table 1 ▸. The structure features a tri-substituted thio­urea mol­ecule with one N atom bearing a benzoyl residue and the other, carrying two hy­droxy­ethyl groups. The thione-S and carbonyl-O atoms lie to the same side of the mol­ecule but are only approximately *syn* as the S1—C1—N2—C6 torsion angle is −49.3 (2)°; the O3—C6—N2—C1 torsion angle is −6.8 (3)°. The hy­droxy­ethyl groups lie to either side of the CN_2_S plane (r.m.s. deviation = 0.018 Å). The O1-hy­droxy­ethyl group is folded toward the thio­amide part of the mol­ecule, an orientation that allows for the formation of an intra­molecular N2—H⋯O1 hydrogen bond that closes an *S*(7) loop, Table 2 ▸. Overall, the mol­ecule is twisted as seen in the dihedral angle of 72.12 (9)° between the CN_2_S atoms and the terminal aryl ring. The C1—N1 bond length is considerably shorter than the C1—N2 bond, which suggests some delocalization of π-electron density over the S1—C1—N1 atoms that does not extend over the C1—N1—C6 atoms, consistent with the large twist about the C1—N2 bond (see above). The bond angles subtended at the C1 and C6 atoms follow the expected trends in that those involving the formally doubly bonded atoms are wider, by approximately 10°, compared with the other angles, Table 1 ▸.

## Gas-phase theoretical structure   

**Figure 2:**
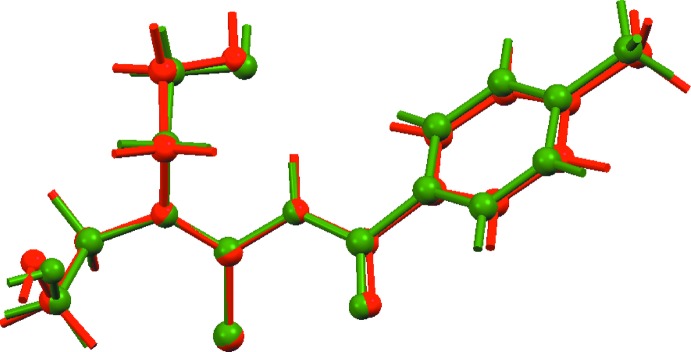
Overlay diagram for experimental (green image) and geometry-optimized (red) mol­ecules for (I). The mol­ecules have been overlapped so the S=C—N—C=O fragments are coincident.

Compound (I) was subjected to gas-phase geometry optimization by long-range corrected wB97XD density functional with Grimme’s D2 dispersion model (Chai & Head-Gordon, 2008 ▸) coupled with Pople’s 6-311+G(*d*,*p*) basis set (Petersson *et al.*, 1988 ▸) as implemented in *Gaussian16* (Frisch *et al.*, 2016 ▸) in order to compare the optimized mol­ecule with the experimental structure. The results of the optimization show that the local minimum structure in the gas-phase was located as confirmed through a frequency analysis with zero imaginary frequency. The superimposition of the experimental and theoretical structures (Macrae *et al.*, 2006 ▸), Fig. 2 ▸, indicates that there are minor differences between the mol­ecules in either phase, with the r.m.s. deviation between them being 0.014 Å. Salient geometric data for the gas-phase structure are included in Table 1 ▸ and correlate very well with the experimental results. The major differences between the experimental and geometry-optimized structures relates to differences in the (i) O3—C6—N2—C1 torsion angles, which deviates further, by approximately 10°, from the *anti*-disposition in the optimized structure, and (ii) N1—C2—C3—O1 and N1—C4—C5—O2 torsion angles, which are disparate, by about 12°, in the experimental structure but are symmetric, *i.e*. ±69°, in the optimized structure.

## Supra­molecular features   

**Figure 3:**
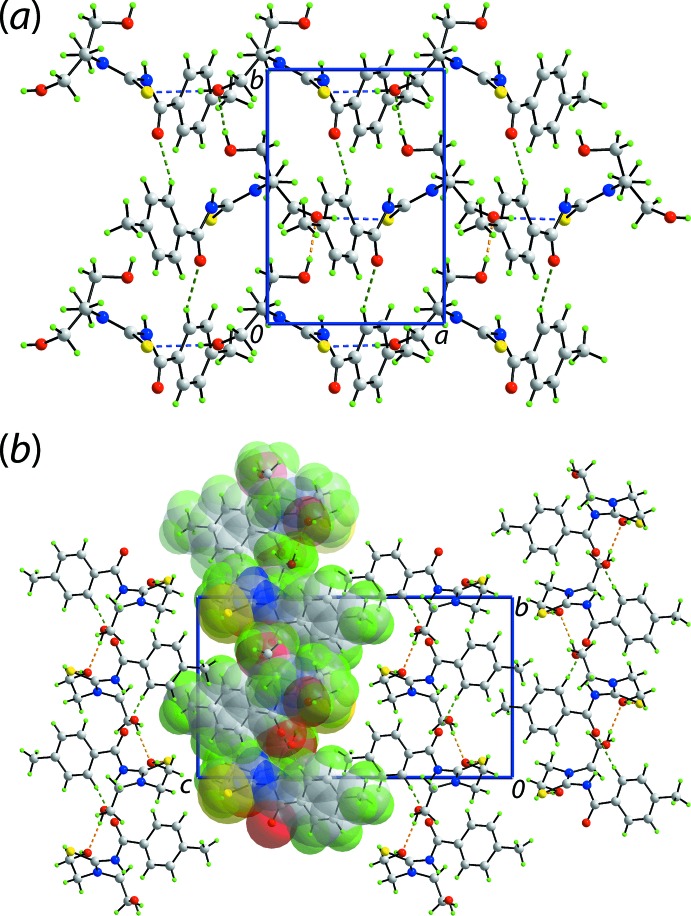
Views of the mol­ecular packing in (I): (*a*) supra­molecular layer in the *ab* plane sustained by hy­droxy-O—H⋯O(hy­droxy), hy­droxy-O—H⋯S(thione) and aryl-C—H⋯O(carbon­yl) inter­actions, and (*b*) view of the unit-cell contents in projection down the *a* axis, highlighting the stacking of layers; one layer is represented in space-filling mode. The O—H⋯O, O—H⋯S and C–H⋯O inter­actions are shown as orange, blue and green dashed lines, respectively.

In the crystal of (I), the O1-hydroxyl group acts as a hydrogen-bond donor to the O2-hy­droxy group, which in turn functions as a donor to the S1-atom, Table 2 ▸. The O—H⋯O hydrogen bonding is propagated by 2_1_ symmetry to generate helical chains along the *b*-axis direction. The O—H⋯S hydrogen bonding serves to connect translationally related chains along the *a*-axis direction and these contacts are reinforced by phenyl-C—H⋯O(carbon­yl) inter­actions. In this way, a supra­molecular layer in the *ab* plane is formed, Fig. 3 ▸(*a*). Layers stack along the *c*-axis direction without directional inter­actions between them, Fig. 3 ▸(*b*).

## Hirshfeld surface analysis   

The calculations of the Hirshfeld surfaces and the two-dimensional fingerprint plots (overall and delineated) for (I) were performed using *Crystal Explorer 17* (Turner *et al.*, 2017 ▸) and published protocols (Tan *et al.*, 2019 ▸).

**Figure 4:**
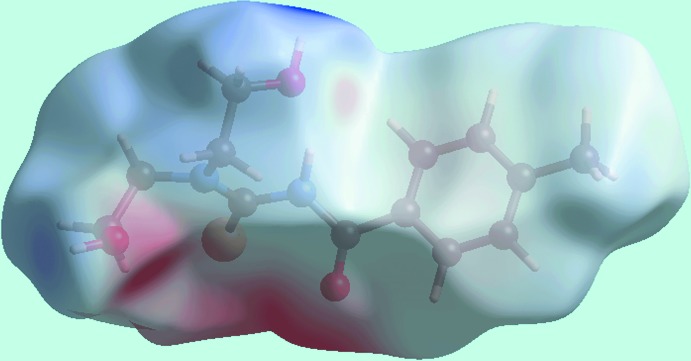
A view of the Hirshfeld surface mapped over the calculated electrostatic potential for (I). The red and blue regions represent negative and positive electrostatic potentials, respectively. The potentials were calculated using the STO-3G basis set at Hartree–Fock level of theory over a range of ±0.18 atomic units.

**Figure 5:**
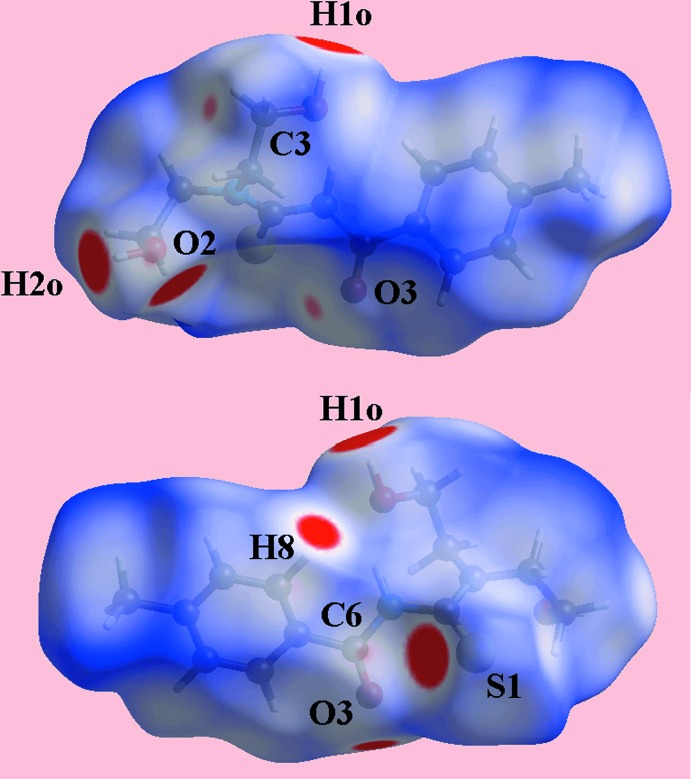
Two views of the Hirshfeld surface mapped over *d*_norm_ for (I) in the range −0.132 to +1.682 arbitrary units.

The Hirshfeld surface mapped over electrostatic potential in Fig. 4 ▸, shows different potentials surrounding the key functional groups. Thus, the donors and acceptors of conventional O—H⋯O and O—H⋯S hydrogen bonds and C—H⋯O contacts appear as blue and red regions, respectively, corresponding to positive and negative potential. The Hirshfeld surface mapped over *d*_norm_ in Fig. 5 ▸ also gives the usual indications of these inter­molecular inter­actions through the appearance of bright-red spots near participating atoms. In addition, short inter­atomic contacts between the hydroxyl-H atom, and carbonyl-C6 and hydroxyl-O2 atoms, and between the ethyl-C5 and hydroxyl-H1*O* atoms, Table 3 ▸, are either characterized as faint-red spots or merged within the bright-red spots corresponding to the conventional hydrogen bonds in Fig. 5 ▸.

The inter­molecular contacts in the crystal of (I) were further analysed using an enrichment ratio (*ER*) descriptor, which is derived from the analysis of the Hirshfeld surface (Jelsch *et al.*, 2014 ▸). The *ER* relates the propensity of pair of chemical species to form a specific inter­action in a crystal. The enrichment ratio, *ER*(*X*, *Y*), for a pair of elements (*X*, *Y*) is defined as the ratio between proportion of actual contacts in the crystal to the theoretical proportion of random contacts. This ratio is greater than unity for a pair of elements having a high likelihood to form contacts in a crystal, while it is less than one for a pair which tends to avoid contacts with each other. A listing of *ER* values for (I) is given in Table 4 ▸. The enrichment ratios greater than unity for the atom pairs (O, H) and (S, H), Table 4 ▸, are consistent with the high propensity for the formation of the O—H⋯O and O—H⋯S hydrogen bonds in the crystal. It is also evident that the value greater than unity for (C, H) arises from the C⋯H/H⋯C contacts.

**Figure 6:**
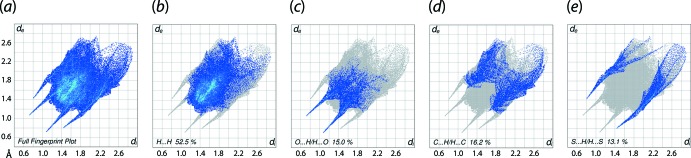
(*a*) A comparison of the full two-dimensional fingerprint plot for (I) and those delineated into (*b*) H⋯H, (*c*) O⋯H/H⋯O, (*d*) C⋯H/H⋯C and (*e*) S⋯H/H⋯S contacts.

The overall fingerprint plots for (I) and those delineated into H⋯H, O⋯H/H⋯O, C⋯H/H⋯C and S⋯H/H⋯S contacts are illustrated in Fig. 6 ▸(*a*)–(*e*), respectively. A summary of the percentage contributions from the various contacts in the crystal are given in Table 5 ▸. The contribution from H⋯H contacts are reflected in the middle of the scattered point and cover the greatest area in the plot, and make the most significant contribution (52.5%) to the total Hirshfeld surface, Fig. 6 ▸(*b*) and has an *ER* value of 0.92, *i.e*. close to unity. The contribution from O⋯H/H⋯O contacts is viewed as long spikes at *d*_e_ + *d*_i_ ∼1.8 Å, with points scattered around different regions in the delineated fingerprint plot, Fig. 6 ▸(*c*). In the fingerprint delineated into C⋯H/H⋯C contacts in Fig. 6 ▸(*d*), a pair of small tips at *d*_e_ + *d*_i_ < 2.8 Å is the result of short inter­atomic contacts, Table 3 ▸, including an inter­layer contact (H13*A*⋯O1). The percentage contribution from S⋯H/H⋯S contacts (13.1%) reflect the presence of O—H⋯S hydrogen bonds and are apparent through the appearance of asymmetric spikes at *d*_e_ + *d*_i_ ∼2.1 Å in Fig. 6 ▸(*e*).

## Computational chemistry   

The inter­molecular O—H⋯O, O—H⋯S and C—H⋯O inter­actions occurring between the respective pairs of mol­ecules were subjected to energy calculations by DFT-wB97XD/aug-cc-pVTZ (Woon & Dunning, 1993 ▸) for the evaluation of the strength of these inter­actions. With reference to the BSSE corrected inter­action energies (*E* ^BSSE^_int_) listed in Table 6 ▸, the O—H⋯O hydrogen bond has the greatest inter­action energy, followed by C—H⋯O and O—H⋯S. Unexpectedly, the C—H⋯O inter­action has an energy approximately 3–4 kcal mol^−1^ more stable than the O—H⋯S inter­action despite phenyl-C—H being a weak hydrogen-bond donor and thione-S a weak acceptor, and that such inter­actions are known to be dispersive in nature (Bhattacharyya *et al.*, 2013 ▸). The donor–acceptor inter­actions were also evaluated by a natural bond orbital (NBO) population analysis (Reed *et al.*, 1988 ▸), which revealed that the net NBO charge for H8⋯O3 is 0.8 compared to 0.6 for H2*O*⋯S1, thereby confirming the relative strength of these inter­actions.

**Figure 7:**
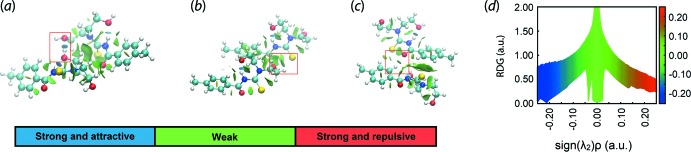
NCI plots for the dimeric aggregates in (I) sustained by (*a*) O—H⋯O, (*b*) O—H⋯S and (*c*) C—H⋯O inter­actions (highlighted in boxes), and (*d*) plot of RDG *versus* sign(λ_2_)ρ(*r*). The gradient cut-off is set at 0.4 and the colour scale is −0.03 < ρ < 0.03 atomic units.

To complement the results of the calculations on the inter­action energies, the dimeric structures were subjected to further analysis by *NCIPLOT* (Johnson *et al.*, 2010 ▸). The analysis provides a convenient visualization index on the strength of any existing non-covalent inter­actions through a red–blue–green colour scheme on the isosurface, *i.e*. red is indicative of a strong repulsive inter­action, blue is indicative of strong attractive inter­action while green is indicative of a weak inter­action (Contreras-García *et al.*, 2011 ▸). The results, illus­trated in Fig. 7 ▸, reveal that the O—H⋯O inter­action is clearly strong and attractive, while both O—H⋯S and C—H⋯O are considered weak inter­actions.

**Figure 8:**
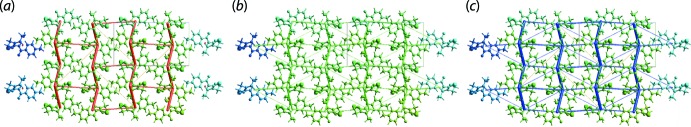
The energy framework diagrams for (I) showing (*a*) *E*_electrostatic_ (red cylinders), (*b*) *E*_dispersion_ (green cylinders) and (*c*) *E*_total_ (blue cylinders), viewed along the *a* axis. The frameworks were adjusted to the same scale factor of 50 with a cut-off value of 2.39 kcal mol^−1^ within 2 × 2 × 2 unit cells. The corresponding cylinder radii are proportional to the relative magnitude of the energies.

As the mol­ecular packing is governed directionally by hydrogen bonding between mol­ecules, the energy frameworks were simulated (Turner *et al.*, 2017 ▸) in order to compare the topology of these inter­molecular inter­actions. A detailed analysis of the energy frameworks shown in Fig. 8 ▸ reveals the crystal of (I) is mainly stabilized by electrostatic and dispersive forces. The total electrostatic energy (*E*_electrostatic_) of all pairwise inter­actions sums to −36.11 kcal mol^−1^, while the total dispersion energy term (*E*_dispersion_) computes to −43.83 kcal mol^−1^.

## Database survey   

The crystal structure of the parent compound, PhC(=O)N(H)C(=S)N(CH_2_CH_2_OH)_2_, (II), has been reported twice (Koch *et al.*, 1995 ▸; Cornejo *et al.*, 2005 ▸; refcodes ZAJWAI and ZAJWAI01, respectively). The conformation of this mol­ecule and that of (I) are very similar and the geometric parameters describing chemically equivalent parameters are generally within experimental errors. The most important conformational difference is seen in the pair of N1—C2—C3—O1 [73.7 (2)°] and N1—C4—C5—O1 [–53.9 (2)°] torsion angles, which span a range of approximately 20° in (II) *cf*. approximately 12° in (I). The mol­ecular packing in (II) also features O—H⋯O hydrogen and O—H⋯S hydrogen bonding, as for (I), leading to a supra­molecular layer; the intra­molecular amine-N—H⋯·O(hy­droxy) hydrogen bond persists. However, in the case of (II), there are directional inter­actions between layers, *i.e*. of the type phenyl-C—H⋯π(phen­yl), to sustain a three-dimensional architecture. The other closely related structure is that of 4-MePhC(=O)N(H)C(=S)N(Me)CH_2_CH_2_OH) (Jamaludin *et al.*, 2016 ▸; refcode GADBOF). Here, the intra­molecular amine-N—H⋯O(hy­droxy) hydrogen bond is also found and the most prominent feature of the mol­ecular packing is the formation of supra­molecular helical chains mediated by hy­droxy-O—H⋯O(carbon­yl) hydrogen bonds.

## Synthesis and crystallization   

All chemicals and solvents were used as purchased without purification. The reactions were carried out under ambient conditions. The melting point was measured using a Hanon MP-450 melting point apparatus. The CHN elemental analysis was performed on a LECO TruSpec Micro analyser under helium atmosphere with glycine being used as the standard. The IR spectrum was measured on a Bruker Vertex 70v FT–IR spectrophotometer from 4000 to 400 cm^−1^. The ^1^H and ^13^C{^1^H} spectra were recorded in DMSO-*d*_6_ solutions on a Bruker Ascend 400 MHz NMR spectrometer with chemical shifts relative to tetra­methyl­silane (TMS). The optical absorption spectra were measured on 10 and 100 µM ethanol:aceto­nitrile (1:1) solutions in the range 190–1100 nm on a double-beam Shimadzu UV 3600 Plus UV–vis spectrophotometer. The thermogravimetric analysis (TGA) was performed on a Perkin Elmer STA 6000 Simultaneous Thermogravimetric Analyzer in the range of 35–900°C under a nitro­gen atmosphere at a flow rate of 10°C min^−1^. The experimental powder X-ray diffraction pattern was measured on a Rigaku MiniFlex diffractometer with Cu Kα_1_ radiation (λ = 1.54056 Å) in the 2θ range of 5–70° and a step size of 0.02°. The experimental PXRD patterns were compared to the simulated PXRD patterns calculated from the CIF using the Rigaku *PDXL* structure analysis software package. The patterns matched indicating that the reported crystal structure is representative of the bulk material.

Synthesis of (I): An excess of thionyl chloride (Merck) was mixed with 4-methyl­benzoic acid (Merck, 1 mmol) and the resulting solution was refluxed until a pale-yellow solution was obtained. The excess thionyl chloride was removed on a water bath, leaving only 4-methyl­benzoyl chloride, which is a yellow, viscous liquid. Ammonium thio­cyanate (Fisher, 1 mmol) was added into an acetone (30 ml) solution of 4-methyl­benzoyl chloride (1 mmol). The solution turned yellow after stirring for 2 h. The white precipitate (ammonium chloride) was isolated upon filtration and to the yellow filtrate, bis­(hy­droxy­eth­yl)amine (Acros, 1 mmol) was carefully added followed by stirring for 1 h. Upon the addition of di­chloro­methane (50 ml), a yellow precipitate was obtained, which was collected by filtration. Recrystallization from its hot acetone solution yielded colourless blocks after slow evaporation. White solid, yield 56%, m.p. 400.3–402.1 K. Elemental analysis: C_13_H_18_N_2_O_3_S, found (calculated): C 55.59 (55.30), H 6.57 (6.43), N 9.79 (9.92). IR (ATR; cm^−1^): 3312 (*br*, νOH), 3158 (*br*, νNH), 3061 (*w*, νCH_aro_), 2955–2881 (*w*, νCH), 1686 (*s*, νC=O), 1539 (*s*, νC=C), 1250 (*s*, νC—N), 1054 (*s*, νC=S), 747 (*s*, δCH). UV (ethanol:aceto­nitrile; 5ml:5ml): λ_max_ nm (assignment; log ∊) 354.4 (*n*→π^*^; 4.34), 294.0 (*n*→π^*^; 4.98), 246.4 (π→π^*^; 5.17), 202.6 (σ→π^*^; 5.17). ^1^H NMR (400 MHz, DMSO-*d*_6_; see Fig. 1 ▸ for the numbering scheme): δ 10.78 (1H, *br*, *s*, NH), 7.76 (2H, *d*, 2-phenyl, ^3^ *J*_HH_ = 7.72 Hz), 7.31 (2H, *d*, 3-phenyl, ^3^ *J*_HH_ = 7.6 Hz), 5.66 (1H, *br*, *s*, OH), 4.87 (1H, *t*, OH, ^3^ *J*_OH–H_ = 5.00 Hz), 3.98 (2H, overlapping *t*, CH_2_–C2, ^3^ *J*_HH_ = 6.24 Hz, ^3^ *J*_HH_ = 6.08 Hz), 3.76 (2H, *m*, CH_2_–C3), 3.70 (4H, *m*, CH_2_–C4, C5), 2.37 (3H, *s*, CH_3_). ^13^C{^1^H} NMR (100 MHz, DMSO-*d*_6_): δ 180.63 (C1), 163.78 (C6), 141.88 (C7), 130.13 (C10), 128.47 (C9, C11), 127.28 (C8, C12), 58.58 (C5), 56.95 (C3), 54.42 (C4), 54.29 (C2), 20.42 (C13).

The pyrolytic processes for (I) was resolved into four main stages. The first stage involves the liberation of H_2_O between 135 and 165°C, which corresponds to approximate 6% of the weight for (I). The second stage between 160 and 240°C is attributed to the loss of a 4-methyl­benzaldehyde fragment, corresponding to 45% weight loss. Subsequently, the remaining fragments undergo further pyrolysis to result in the liberation of ethanol (31% weight) and ammonia (17–18%) in the range 230 to 300°C and 300°C onward, respectively. Compound (I) decomposed at temperatures beyond 700°C.

## Refinement   

Crystal data, data collection and structure refinement details are summarized in Table 7 ▸. Carbon-bound H atoms were placed in calculated positions (C—H = 0.93–0.97 Å) and were included in the refinement in the riding-model approximation, with *U*_iso_(H) set to 1.2–1.5*U*_eq_(C). The O- and N-bound H atoms were located from a difference map and refined with O—H and N—H = 0.84±0.01 and 0.88±0.01 Å, respectively, and with *U*_iso_(H) = 1.5U_eq_(O) and 1.2*U*_eq_(N).

## Supplementary Material

Crystal structure: contains datablock(s) I, global. DOI: 10.1107/S2056989019012581/hb7854sup1.cif

Structure factors: contains datablock(s) I. DOI: 10.1107/S2056989019012581/hb7854Isup2.hkl

CCDC reference: 1919878

Additional supporting information:  crystallographic information; 3D view; checkCIF report

## Figures and Tables

**Table 1:** Selected geometric parameters for (I) determined experimentally (X-ray) and from theory

Parameter	X-ray	Theory
C1—S1	1.6744 (17)	1.671
C1—N1	1.335 (2)	1.368
C1—N2	1.396 (2)	1.404
C6—O3	1.214 (2)	1.220
C6—N2	1.382 (2)	1.396
S1—C1—N1	123.97 (13)	124.2
S1—C1—N2	121.67 (12)	122.2
N1—C1—N2	114.30 (14)	113.5
O3—C6—N2	122.09 (17)	123.3
O3—C6—C7	122.23 (16)	122.0
N2—C6—C7	115.64 (15)	114.7
S1—C1—N2—C6	−49.3 (2)	−43.5
S1—C1—N1—C2	171.63 (12)	167.1
S1—C1—N1—C4	−7.3 (2)	−7.6
O3—C6—N2—C1	−6.8 (3)	−18.2
O3—C6—C7—C8	160.25 (17)	156.2
N1—C2—C3—O1	−70.1 (2)	−69.2
N1—C4—C5—O2	57.5 (2)	69.0

**Table 2:** Hydrogen-bond geometry (Å, °)

*D*—H⋯*A*	*D*—H	H⋯*A*	*D*⋯*A*	*D*—H⋯*A*
N2—H2*N*⋯O1	0.87 (1)	1.91 (1)	2.728 (2)	157 (1)
O1—H1*O*⋯O2^i^	0.83 (2)	1.94 (2)	2.769 (2)	172 (2)
O2—H2*O*⋯S1^ii^	0.84 (2)	2.38 (2)	3.2049 (14)	171 (2)
C8—H8⋯O3^iii^	0.93	2.38	3.251 (2)	156

Symmetry codes: (i) 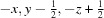; (ii) 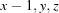; (iii) 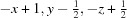.

**Table 3:** Summary of short inter­atomic contacts (Å) in (I) The inter­atomic distances are calculated in *Crystal Explorer 17* (Turner *et al.*, 2017 ▸) whereby the *X*—H bond lengths are adjusted to their neutron values.

Contact	Distance	Symmetry operation
H1*O*⋯H2*O*	2.26	−*x*, − 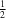 + *y*, − 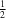 − *z*
C3⋯O3	3.112 (2)	−*x*, − 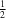 + *y*, 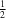 − *z*
H2*B*⋯O3	2.58	−*x*, − 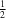 + *y*, 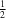 − *z*
H3*B*⋯O3	2.69	−*x*, − 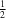 + *y*, 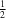 − *z*
C5⋯H1*O*	2.73	−*x*, − 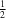 + *y*, 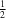 − *z*
H13*A*⋯O1	2.67	1 − *x*, 1 − *y*, −*z*
C6⋯O2	3.177 (2)	1 + *x*, *y*, *z*
C8⋯H2*B*	2.78	1 + *x*, *y*, *z*

**Table 4:** Enrichment ratios for (I)

Parameter	Ratio
H⋯H	0.92
C⋯H	1.21
O⋯H	1.21
S⋯H	1.33
C⋯O	0.54

**Table 5:** Percentage contributions of inter­atomic contacts to the Hirshfeld surface for (I)

Contact	Percentage contribution
H⋯H	52.5
C⋯H/H⋯C	16.2
O⋯H/H⋯O	15.0
S⋯H/H⋯S	13.1
N⋯H/H⋯N	1.5
C⋯C	0.3
C⋯O/O⋯C	0.8
N⋯O/O⋯N	0.1
O⋯O	0.3
C⋯N/N⋯C	0.2

**Table 6:** Summary of inter­action energies (kcal mol^−1^) calculated for several directional contacts in (I)

Contact	*E*_tot_
O1—H1*O*⋯O2	−14.52
O2—H2*O*⋯S1	−6.27
C8—H8⋯O3	−9.65

**Table 7:** Experimental details

Crystal data	
Chemical formula	C_13_H_18_N_2_O_3_S
*M*_r_	282.35
Crystal system, space group	Monoclinic, *P*2_1_/*c*
Temperature (K)	293
*a*, *b*, *c* (Å)	7.4051 (10), 10.6213 (15), 18.569 (3)
β (°)	94.117 (2)
*V* (Å^3^)	1456.7 (4)
*Z*	4
Radiation type	Mo *K*α
μ (mm^−1^)	0.23
Crystal size (mm)	0.12 × 0.09 × 0.08

Data collection	
Diffractometer	Bruker SMART APEX
Absorption correction	Multi-scan (*SADABS*; Sheldrick, 1996 ▸)
*T*_min_, *T*_max_	0.655, 0.746
No. of measured, independent and observed [*I* > 2σ(*I*)] reflections	18125, 3339, 2263
*R*_int_	0.051
(sin θ/λ)_max_ (Å^−1^)	0.650

Refinement	
*R*[*F* ^2^ > 2σ(*F* ^2^)], *wR*(*F* ^2^), *S*	0.039, 0.098, 1.04
No. of reflections	3339
No. of parameters	182
No. of restraints	3
H-atom treatment	H atoms treated by a mixture of independent and constrained refinement
Δρ_max_, Δρ_min_ (e Å^−3^)	0.17, −0.20

Computer programs: *SMART* and *SAINT* (Bruker, 2008 ▸), *SHELXS97* (Sheldrick, 2008 ▸), *SHELXL2014/7* (Sheldrick, 2015 ▸), *ORTEP-3 for Windows* (Farrugia, 2012 ▸), *DIAMOND* (Brandenburg, 2006 ▸) and *publCIF* (Westrip, 2010 ▸).

## supplementary crystallographic information

### Crystal data

**Table:**

C_13_H_18_N_2_O_3_S	*F*(000) = 600
*M**_r_* = 282.35	*D*_x_ = 1.287 Mg m^−^^3^
Monoclinic, *P*2_1_/*c*	Mo *K*α radiation, λ = 0.71073 Å
*a* = 7.4051 (10) Å	Cell parameters from 2509 reflections
*b* = 10.6213 (15) Å	θ = 2.2–22.9°
*c* = 18.569 (3) Å	µ = 0.23 mm^−^^1^
β = 94.117 (2)°	*T* = 293 K
*V* = 1456.7 (4) Å^3^	Prism, colourless
*Z* = 4	0.12 × 0.09 × 0.08 mm

### Data collection

**Table:**

Bruker SMART APEX diffractometer	3339 independent reflections
Radiation source: fine-focus sealed tube	2263 reflections with *I* > 2σ(*I*)
Graphite monochromator	*R*_int_ = 0.051
φ and ω scans	θ_max_ = 27.5°, θ_min_ = 2.2°
Absorption correction: multi-scan (SADABS; Sheldrick, 1996)	*h* = −9→9
*T*_min_ = 0.655, *T*_max_ = 0.746	*k* = −13→13
18125 measured reflections	*l* = −24→24

### Refinement

**Table:**

Refinement on *F*^2^	Primary atom site location: structure-invariant direct methods
Least-squares matrix: full	Secondary atom site location: difference Fourier map
*R*[*F*^2^ > 2σ(*F*^2^)] = 0.039	Hydrogen site location: mixed
*wR*(*F*^2^) = 0.098	H atoms treated by a mixture of independent and constrained refinement
*S* = 1.03	*w* = 1/[σ^2^(*F*_o_^2^) + (0.0374*P*)^2^ + 0.2262*P*] where *P* = (*F*_o_^2^ + 2*F*_c_^2^)/3
3339 reflections	(Δ/σ)_max_ < 0.001
182 parameters	Δρ_max_ = 0.17 e Å^−^^3^
3 restraints	Δρ_min_ = −0.20 e Å^−^^3^

### Special details

**Table:**

Geometry. All esds (except the esd in the dihedral angle between two l.s. planes) are estimated using the full covariance matrix. The cell esds are taken into account individually in the estimation of esds in distances, angles and torsion angles; correlations between esds in cell parameters are only used when they are defined by crystal symmetry. An approximate (isotropic) treatment of cell esds is used for estimating esds involving l.s. planes.

### Fractional atomic coordinates and isotropic or equivalent isotropic displacement parameters (Å^2^)

**Table:**

	*x*	*y*	*z*	*U*_iso_*/*U*_eq_	
S1	0.32019 (6)	0.58997 (5)	0.40517 (3)	0.05150 (16)	
O1	0.20850 (18)	0.31841 (13)	0.20234 (8)	0.0600 (4)	
H1O	0.239 (3)	0.2473 (13)	0.1888 (13)	0.090*	
O2	−0.27874 (16)	0.58125 (13)	0.35097 (7)	0.0534 (4)	
H2O	−0.3778 (19)	0.585 (2)	0.3699 (11)	0.080*	
O3	0.37968 (18)	0.75187 (12)	0.26514 (8)	0.0602 (4)	
N1	0.06795 (17)	0.47500 (13)	0.31953 (7)	0.0382 (3)	
N2	0.31599 (19)	0.54248 (13)	0.26306 (8)	0.0402 (3)	
H2N	0.304 (2)	0.4767 (12)	0.2356 (8)	0.048*	
C1	0.2270 (2)	0.53452 (15)	0.32668 (9)	0.0380 (4)	
C2	−0.0239 (2)	0.44158 (17)	0.24885 (10)	0.0448 (4)	
H2A	0.0053	0.5045	0.2137	0.054*	
H2B	−0.1537	0.4438	0.2528	0.054*	
C3	0.0273 (2)	0.31392 (18)	0.22177 (11)	0.0524 (5)	
H3A	0.0155	0.2513	0.2592	0.063*	
H3B	−0.0526	0.2907	0.1802	0.063*	
C4	−0.0291 (2)	0.44025 (17)	0.38245 (10)	0.0456 (4)	
H4A	0.0583	0.4179	0.4218	0.055*	
H4B	−0.1024	0.3664	0.3707	0.055*	
C5	−0.1497 (2)	0.54346 (19)	0.40748 (10)	0.0497 (5)	
H5A	−0.2125	0.5138	0.4483	0.060*	
H5B	−0.0762	0.6153	0.4232	0.060*	
C6	0.3952 (2)	0.64952 (16)	0.23710 (9)	0.0405 (4)	
C7	0.4941 (2)	0.63211 (16)	0.17111 (9)	0.0393 (4)	
C8	0.5608 (2)	0.51755 (17)	0.14953 (9)	0.0449 (4)	
H8	0.5419	0.4455	0.1764	0.054*	
C9	0.6557 (3)	0.5097 (2)	0.08809 (10)	0.0562 (5)	
H9	0.7011	0.4322	0.0747	0.067*	
C10	0.6843 (3)	0.6145 (2)	0.04626 (10)	0.0584 (5)	
C11	0.6157 (3)	0.7282 (2)	0.06798 (11)	0.0631 (6)	
H11	0.6320	0.7999	0.0404	0.076*	
C12	0.5241 (3)	0.73742 (18)	0.12945 (11)	0.0557 (5)	
H12	0.4815	0.8154	0.1434	0.067*	
C13	0.7870 (4)	0.6055 (3)	−0.02119 (12)	0.0919 (9)	
H13A	0.7224	0.6506	−0.0598	0.138*	
H13B	0.7983	0.5187	−0.0346	0.138*	
H13C	0.9052	0.6416	−0.0120	0.138*	

### Atomic displacement parameters (Å^2^)

**Table:**

	*U*^11^	*U*^22^	*U*^33^	*U*^12^	*U*^13^	*U*^23^
S1	0.0407 (3)	0.0703 (4)	0.0435 (3)	−0.0051 (2)	0.00312 (19)	−0.0119 (2)
O1	0.0472 (8)	0.0542 (9)	0.0806 (10)	−0.0075 (7)	0.0181 (7)	−0.0248 (7)
O2	0.0382 (7)	0.0691 (9)	0.0540 (8)	0.0084 (6)	0.0118 (6)	0.0126 (7)
O3	0.0621 (9)	0.0393 (8)	0.0818 (10)	−0.0049 (6)	0.0227 (7)	−0.0135 (7)
N1	0.0332 (7)	0.0397 (8)	0.0423 (8)	−0.0014 (6)	0.0059 (6)	−0.0025 (6)
N2	0.0438 (8)	0.0378 (8)	0.0399 (8)	−0.0097 (7)	0.0093 (6)	−0.0063 (6)
C1	0.0357 (9)	0.0356 (9)	0.0432 (10)	0.0024 (7)	0.0054 (7)	−0.0012 (7)
C2	0.0370 (9)	0.0481 (11)	0.0487 (10)	−0.0027 (8)	−0.0003 (8)	−0.0064 (8)
C3	0.0458 (11)	0.0512 (12)	0.0613 (12)	−0.0102 (9)	0.0114 (9)	−0.0150 (9)
C4	0.0400 (10)	0.0482 (11)	0.0491 (11)	−0.0029 (8)	0.0068 (8)	0.0090 (8)
C5	0.0430 (10)	0.0635 (12)	0.0435 (10)	−0.0005 (9)	0.0097 (8)	0.0033 (9)
C6	0.0346 (9)	0.0376 (10)	0.0491 (10)	−0.0020 (7)	0.0019 (8)	−0.0023 (8)
C7	0.0356 (9)	0.0400 (10)	0.0419 (9)	−0.0061 (7)	−0.0003 (7)	0.0023 (7)
C8	0.0481 (10)	0.0430 (10)	0.0443 (10)	−0.0006 (8)	0.0073 (8)	0.0073 (8)
C9	0.0598 (13)	0.0569 (13)	0.0530 (12)	0.0013 (10)	0.0121 (10)	−0.0038 (10)
C10	0.0558 (12)	0.0773 (16)	0.0425 (11)	−0.0167 (11)	0.0067 (9)	0.0046 (10)
C11	0.0703 (14)	0.0617 (14)	0.0577 (13)	−0.0171 (11)	0.0080 (11)	0.0206 (11)
C12	0.0600 (12)	0.0422 (11)	0.0653 (13)	−0.0075 (9)	0.0076 (10)	0.0085 (9)
C13	0.0952 (19)	0.126 (2)	0.0581 (14)	−0.0220 (17)	0.0303 (14)	0.0050 (14)

### Geometric parameters (Å, º)

**Table:**

S1—C1	1.6744 (17)	C4—H4B	0.9700
O1—C3	1.415 (2)	C5—H5A	0.9700
O1—H1O	0.832 (9)	C5—H5B	0.9700
O2—C5	1.425 (2)	C6—C7	1.483 (2)
O2—H2O	0.836 (9)	C7—C8	1.383 (2)
O3—C6	1.214 (2)	C7—C12	1.387 (2)
N1—C1	1.335 (2)	C8—C9	1.384 (2)
N1—C4	1.462 (2)	C8—H8	0.9300
N1—C2	1.477 (2)	C9—C10	1.382 (3)
N2—C6	1.382 (2)	C9—H9	0.9300
N2—C1	1.396 (2)	C10—C11	1.382 (3)
N2—H2N	0.866 (9)	C10—C13	1.514 (3)
C2—C3	1.504 (2)	C11—C12	1.373 (3)
C2—H2A	0.9700	C11—H11	0.9300
C2—H2B	0.9700	C12—H12	0.9300
C3—H3A	0.9700	C13—H13A	0.9600
C3—H3B	0.9700	C13—H13B	0.9600
C4—C5	1.508 (3)	C13—H13C	0.9600
C4—H4A	0.9700		
			
C3—O1—H1O	109.1 (18)	C4—C5—H5A	109.4
C5—O2—H2O	105.3 (16)	O2—C5—H5B	109.4
C1—N1—C4	121.39 (14)	C4—C5—H5B	109.4
C1—N1—C2	123.27 (14)	H5A—C5—H5B	108.0
C4—N1—C2	115.33 (13)	O3—C6—N2	122.09 (17)
C6—N2—C1	125.67 (14)	O3—C6—C7	122.23 (16)
C6—N2—H2N	119.0 (12)	N2—C6—C7	115.64 (15)
C1—N2—H2N	114.6 (12)	C8—C7—C12	118.21 (17)
N1—C1—N2	114.30 (14)	C8—C7—C6	123.83 (16)
N1—C1—S1	123.97 (13)	C12—C7—C6	117.95 (16)
N2—C1—S1	121.67 (12)	C7—C8—C9	120.32 (17)
N1—C2—C3	113.76 (15)	C7—C8—H8	119.8
N1—C2—H2A	108.8	C9—C8—H8	119.8
C3—C2—H2A	108.8	C10—C9—C8	121.45 (19)
N1—C2—H2B	108.8	C10—C9—H9	119.3
C3—C2—H2B	108.8	C8—C9—H9	119.3
H2A—C2—H2B	107.7	C11—C10—C9	117.76 (18)
O1—C3—C2	108.71 (15)	C11—C10—C13	120.9 (2)
O1—C3—H3A	109.9	C9—C10—C13	121.4 (2)
C2—C3—H3A	109.9	C12—C11—C10	121.24 (19)
O1—C3—H3B	109.9	C12—C11—H11	119.4
C2—C3—H3B	109.9	C10—C11—H11	119.4
H3A—C3—H3B	108.3	C11—C12—C7	121.01 (19)
N1—C4—C5	113.48 (14)	C11—C12—H12	119.5
N1—C4—H4A	108.9	C7—C12—H12	119.5
C5—C4—H4A	108.9	C10—C13—H13A	109.5
N1—C4—H4B	108.9	C10—C13—H13B	109.5
C5—C4—H4B	108.9	H13A—C13—H13B	109.5
H4A—C4—H4B	107.7	C10—C13—H13C	109.5
O2—C5—C4	111.00 (15)	H13A—C13—H13C	109.5
O2—C5—H5A	109.4	H13B—C13—H13C	109.5
			
C4—N1—C1—N2	169.93 (14)	O3—C6—C7—C8	160.25 (17)
C2—N1—C1—N2	−11.1 (2)	N2—C6—C7—C8	−22.1 (2)
C4—N1—C1—S1	−7.3 (2)	O3—C6—C7—C12	−18.5 (3)
C2—N1—C1—S1	171.63 (12)	N2—C6—C7—C12	159.18 (16)
C6—N2—C1—N1	133.37 (17)	C12—C7—C8—C9	0.3 (3)
C6—N2—C1—S1	−49.3 (2)	C6—C7—C8—C9	−178.41 (16)
C1—N1—C2—C3	89.8 (2)	C7—C8—C9—C10	−0.9 (3)
C4—N1—C2—C3	−91.18 (18)	C8—C9—C10—C11	0.4 (3)
N1—C2—C3—O1	−70.1 (2)	C8—C9—C10—C13	−179.6 (2)
C1—N1—C4—C5	86.72 (19)	C9—C10—C11—C12	0.7 (3)
C2—N1—C4—C5	−92.28 (18)	C13—C10—C11—C12	−179.3 (2)
N1—C4—C5—O2	57.5 (2)	C10—C11—C12—C7	−1.3 (3)
C1—N2—C6—O3	−6.8 (3)	C8—C7—C12—C11	0.7 (3)
C1—N2—C6—C7	175.48 (15)	C6—C7—C12—C11	179.56 (18)

### Hydrogen-bond geometry (Å, º)

**Table:**

*D*—H···*A*	*D*—H	H···*A*	*D*···*A*	*D*—H···*A*
N2—H2*N*···O1	0.87 (1)	1.91 (1)	2.728 (2)	157 (1)
O1—H1*O*···O2^i^	0.83 (2)	1.94 (2)	2.769 (2)	172 (2)
O2—H2*O*···S1^ii^	0.84 (2)	2.38 (2)	3.2049 (14)	171 (2)
C8—H8···O3^iii^	0.93	2.38	3.251 (2)	156

Symmetry codes: (i) −*x*, *y*−1/2, −*z*+1/2; (ii) *x*−1, *y*, *z*; (iii) −*x*+1, *y*−1/2, −*z*+1/2.
